# Ground reaction force in basketball cutting maneuvers with and without ankle bracing and taping

**DOI:** 10.1590/S1516-31802006000500002

**Published:** 2006-09-07

**Authors:** Isabel de Camargo Neves Sacco, Henrique Yuji Takahasi, Eneida Yuri Suda, Linamara Rizzo Battistella, Cristianne Akie Kavamoto, José Augusto Fernandes Lopes, Jeane Cintra Peixoto de Vasconcelos

**Keywords:** Biomechanics, Ankle, Movement, Kinetics, Motor activity, Basketball, Biomecânica, Tornozelo, Cinética, Atividade motora, Movimento, Basquetebol

## Abstract

**CONTEXT AND OBJECTIVE:**

In basketball, the most common injuries are ankle sprains. For this reason, players frequently use external ankle devices or taping as prophylactic and rehabilitation measures. The purpose of this study was to evaluate ground reaction force (GRF) responses in basketball players while performing typical cutting maneuvers with and without ankle bracing and ankle taping.

**DESIGN AND SETTING:**

Comparative study with experimental design of single-group repeated measurements, at Medical Rehabilitation Division, Hospital das Clínicas, Faculdade de Medicina, Universidade de São Paulo.

**METHODS:**

Vertical (Fy) and medial-lateral (Fz) GRF measurements were made under three conditions (taping, *Aircast*-type orthosis and basketball shoes alone), with analysis of peak forces at foot contact (Fymax1, Fzmax1, Fymax2 and Fzmax2), growth gradient (peak/time) (GG Fymax1, GG Fzmax1, GG Fymax2 and GG Fzmax2) and impulse after foot contact.

**RESULTS:**

Bracing significantly reduced Fymax2 and GG Fymax2. GG Fzmax1 was significantly higher for the sport shoe condition than for the taping condition. Taping increased Fy in relation to the sport shoe at foot contact, but over a longer time interval, without increasing excessive ankle loading. Fz reached a peak in less time, which might generate greater inversion/eversion loading on a player's foot. The *Aircast* exerted better shock-absorbing effect than did the other two conditions, since it generated less vertical force over longer time intervals and smaller medial-lateral forces in relation to taping.

**CONCLUSIONS:**

Ankle bracing and ankle taping action mechanisms are still unclear and therefore should be carefully prescribed. More studies are needed to clarify taping and bracing effects on sporting activities.

## RESUMO

**CONTEXTO E OBJETIVO:**

A lesão mais comum no basquetebol é a entorse de tornozelo. Assim, os atletas freqüentemente utilizam suportes externos como medidas profiláticas. O objetivo deste estudo é avaliar as respostas da força de reação do solo (FRS) durante a execução do movimento de *cutting* do basquetebol com e sem acessórios de tornozelo.

**TIPO DE ESTUDO E LOCAL:**

Estudo comparativo. Delineamento experimental de grupo único com medidas repetidas; Divisão de Medicina de Reabilitação, Hospital das Clínicas, Faculdade de Medicina da Universidade de São Paulo, Brasil.

**MÉTODOS:**

Foram colhidas as forças vertical (Fy) e médio-lateral (Fz) em três condições (bandagem, *Aircast* e calçado esportivo) e analisados os picos de força e de propulsão no contato com o solo (Fymax1, Fzmax1, Fymax2 e Fzmax2), o gradiente de crescimento (pico/tempo) (GC Fymax1, GC Fzmax1, GC Fymax2 e GC Fzmax2) e o impulso após o contato.

**RESULTADOS:**

Os acessórios reduziram significativamente Fymax2 e GC Fymax2. GC FZmax1 foi maior na situação com tênis quando comparado com bandagem. No momento do impacto, a bandagem aumentou a Fy em relação ao calçado, mas em um intervalo de tempo maior, não aumentando a carga articular. Fz atingiu um pico em menor tempo, podendo gerar maior carga eversora/inversora. O *Aircast* exerceu um melhor efeito de absorção de impacto, pois gera menor Fy em um maior intervalo de tempo.

**CONCLUSÕES:**

Apesar do uso freqüente deste tipo de recurso pelos atletas, seu mecanismo de ação ainda é confuso. Mais estudos são necessários para esclarecer seus efeitos nas atividades esportivas a longo prazo.

## INTRODUCTION

Sport injuries are one of the most common types of injury in modern Western societies. Their treatment is not only difficult, but also costly and lengthy. Therefore, the use of preventive measures is justified, from both an economic and a medical standpoint.^1^ For such prevention to be effective, the etiology, risk factors and main injury mechanisms need to be understood.

For basketball players, the ankle is the segment most frequently injured, and inversion sprains present the highest injury incidence rate. The ankle injury rate is 3.85 per 100 participations, with approximately half (45.9%) of the players kept away from competition for a week or more.^2^ It is common to observe players using prophylactic measures, such as taping, wrapping, orthoses and other measures, either as sprain prevention or as rehabilitation.

A large number of studies have investigated the use of taping and orthoses as a prophylactic measure, usually with results defending the stance that bracing usage reduces injuries. However, its exact mechanism of action is still not well understood. Several studies in the literature have described external devices and taping, but with contradictory results. A few studies have investigated the role of proprioception and awareness of foot position in taping and orthoses for sprain prevention. A variety of methods were used by these authors, ranging from kinematics and electromyographic analyses to postural oscillation and stimulation of anesthetized cutaneous receptors of the lateral ligament complex.^3^

Some studies have analyzed the effects of orthoses and taping on athletic performance. Some of these found that external devices or taping impair activities such as jumping and running.^4,5^ One other study found that performance was improved among individuals with ankle instability by using semi-rigid orthoses,^6^ while in a study by Verbrugge^7^ these neither impaired nor improved athletic performance.

Electromyographic studies on the use of external devices and taping also describe different results. Some observed higher response latency or lower electromyographic (EMG) activity in fibular muscles while using orthoses and taping.^8,9^ Others found an improvement in reaction time or higher EMG activity in fibular muscles while using bracing.^10,11^

According to Canavan,^12^ taping and orthosis use by healthy athletes is unjustified. However, an external device or taping is recommended for competition-level athletes with a history of ankle injuries. This provides comfort and helps to control edema during the acute stage of a sprain.

Reductions in the injury rate may result from decreased muscle reaction time for the fibularis brevis in unstable ankles^10^ and also for ankle mechanoreceptor stimulations.^6^ The proprioceptive role of taping and orthoses seems to be greater than their restriction of total movement amplitude in ankles.^6,10^

There are few studies relating to ground reaction force (GRF) and external ankle devices or taping. Most of them studied movements that were less dynamic and functional, and did not find any differences in temporal or magnitude characteristics of GRF.^9,13,14^ One study found that the time to reach vertical GRF under orthosis and taping conditions was shorter than under control conditions during landing.^15^

Caulfield and Garrett^16^ examined the changes in timing and magnitudes of forces sustained by unstable ankles during jump landing, compared with healthy controls. They suggested that the changes in lateral and anterior peak forces observed are likely to result in repeated injury due to significant increases in stress on ankle joint structures, since the peak forces occurred significantly earlier in subjects with functional ankle instability.

In basketball, cutting maneuvers (quick eluding movements) are the mechanism responsible for approximately 30% of sprains.^2^ It has been suggested that when subjects perform cutting tasks during walking and running, the braking forces (anterior/posterior ground reaction force) increase during early stance. The higher braking forces are explained by the need to decelerate in preparation to cut toward the new plane of progression. These greater braking forces have been associated with a relative increase in quadriceps activation during the early stance of cutting tasks.^17^ However, the GRF patterns during cutting activities in subjects wearing ankle braces or taping have not been described to date.

## OBJECTIVE AND HYPOTHESIS

The purpose of this study was to evaluate the dynamic GRF responses among professional basketball players while performing cutting maneuvers under controlled conditions: only using the sport shoes normally used for playing basketball and under two conditions of ankle support (taping and *Aircast*-type orthoses).

If ankle bracing or taping alters ankle stability, it would be expected that changes would occur in the timing and magnitudes of the forces sustained by the braced or taped ankle.

Our hypothesis was that the use of braces or taping would alter the vertical and mediallateral components of GRF during the impact and propulsion phases of basketball cutting maneuvers, and the temporal components of GRF, expressed as growth gradient (peak/time) for these forces and the impulses at 50 ms and 75 ms after foot contact on the platform.

## METHODS

The sample was made up of a group of eight men who had played basketball for at least five years. At the time of the evaluation, they were between the ages of 17 and 25, healthy and without musculoskeletal or joint injuries or any functional or mechanical ankle instabilities. By means of an informed consent agreement, the subjects were made aware of the stages of the experimental protocol, which they read and through which they agreed to participate in the research. The protocol was assessed and approved by the Ethics Committee of the Physical Therapy, Speech and Occupational Therapy Department of the School of Medicine of Universidade de São Paulo.

The experimental protocol was developed at the Gait Laboratory of the Vergueiro Medical Rehabilitation Division and it consisted of two stages.

### Players' characteristics

The players were evaluated by a physical therapist by means of clinical and functional tests on their ankles to verify the absence of mechanical or functional instability.^18-23^ Individuals with well-characterized complaints, either from the interview or functional testing, were considered to present functional instability. The functional test consisted of going down a staircase of 44 stairs; each stair was 18 cm long and 22 cm deep. The subject was asked to go down the staircase once, one step at a time with full contact of the foot shoe sole on the stairs. The time taken to go down the staircase was recorded using a chronometer. The results were classified as less than 18 seconds for the best results, from 18 to 20 seconds for the middle range, and over 20 seconds for the group with the worst results.^24^ Players going downstairs in over 20 s were not included in the study.

### Bracing and taping

The ankle bracing used in this study was the *Aircast Stirrup*-type orthosis (*Aircast Inc.*). This bracing device was chosen because basketball players frequently use it.^5^ The *Aircast* orthosis consists of two semi-rigid thermoplastic structures with a pre-inflated air cell of adjustable position and air volume. The orthosis surrounds the medial and lateral malleoli, reaching up to approximately six inches above the ankle joint and is fastened by Velcro^®^ straps.

**Figure 1:**
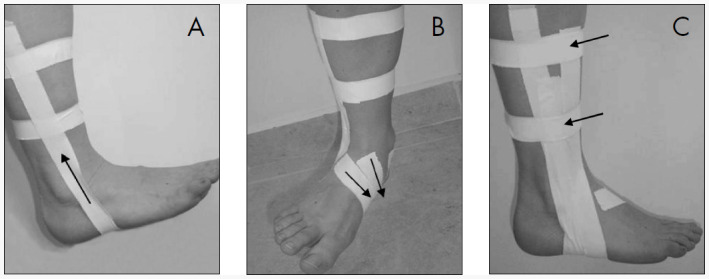
Schematic representation of taping technique. (A) Two strips of adhesive tape around the ankle about 5 and 10 centimeters above the lateral and medial malleoli were attached, and another tape strip was placed, passing over the medial malleolus, heel and lateral malleolus and attaching the tips of this tape to the support base, while maintaining the ankle in dorsiflexion and eversion. (B) Another two tape strips were applied to the instep, passing diagonally around the mid-foot, while also maintaining the ankle in dorsiflexion and eversion. A further strip was also placed across the instep, passing around the mid-foot, and over the lateral malleolus, and attaching it to the support base. (C) Another two tape strips were applied over the two support bases.

The taping technique consisted of applying non-elastic adhesive tape over the individual's skin. This technique has been considered to be the most efficient for joint stabilization.^25^ There are many ways of applying the tape. The technique used in the present study is illustrated in Figure 1. It consisted of, firstly, using two strips of adhesive tape around the ankle about 5 to 10 centimeters above the lateral and medial malleoli, and this was used as a support base. Then another tape strip was placed, passing over the medial malleolus, heel and lateral malleolus and attaching the tips of this tape to the support base, while maintaining the ankle in dorsiflexion and eversion. Another two tape strips were applied to the instep, passing diagonally around the mid-foot, while also maintaining the ankle in dorsiflexion and eversion. A further strip was also placed across the instep, passing around the mid-foot, and over the lateral malleolus, and attaching it to the support base. Finally, another two tape strips were applied over the two support bases.

During these trials, the sport shoes used by the subjects were those usually worn for playing basketball. The use of these sport shoes alone formed the control group (one individual was his own control).

### Dynamics

During the second stage of the experimental protocol, the basketball cutting maneuver was dynamically evaluated. Cutting was chosen because it is the mechanism responsible for approximately 30% of sprains.^2^ This maneuver was performed by the players with and without the use of bracing or taping and under the control conditions of only using the normal sport shoes. The study design was as follows. The subjects were evaluated while performing cutting maneuvers on the force platform, and the change in direction was carried out using each player's dominant lower limb. The cutting movement was tested under each of these three conditions: *Aircast*-type bracing, taping and sport shoes alone. Each of these three conditions was repeated five times (five attempts), and the mean was calculated from these results.

**Figure 2:**
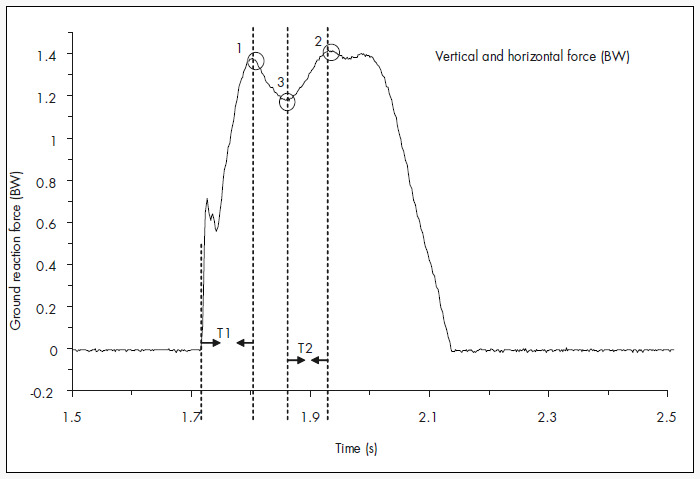
Graphic representation of vertical and horizontal forces during cutting maneuver with basketball shoes: (1) Fymax1 and Fzmax1, maximum vertical and medial-lateral forces at foot contact on the ground; (2) Fymax2 and Fzmax2, maximum vertical and medial-lateral forces at the moment of propulsion; (3) Fymin and Fzmin, minimum vertical and medial-lateral forces; (T1) time to reach Fymax1 or Fzmax1, (T2) time to reach Fymax2 or Fzmax2. BW = body weight.

This study used a force platform made by Applied Marine Technology, Inc., for acquisition and analysis of the vertical and anteroposterior and medial-lateral horizontal components of the ground reaction force. This platform was placed at ground level in a room with approximately 20 linear meters for the locomotion movements. Data were collected during the five attempts, each one with a sampling frequency of 500 Hz for a period of six seconds, which was compatible with these types of movements.^26^ Table 1 and Figure 2 describe the vertical and medial-lateral ground reaction force variables analyzed during this movement.

The vertical and medial-lateral ground reaction force variables for each study condition were normalized for the body weight of each subject and then filtered using a low-pass Butterworth filter with a 200 Hz cutoff frequency, as suggested by Roesler et al.^26^

**Table 1:** Description of the vertical and horizontal ground reaction force variables in cutting movements in basketball players

Movement	Symbol	Description
**Cutting maneuver**	**Fymax1 and Fzmax1**	Maximum vertical and medial-lateral forces at foot contact on the ground
	**Fymax2 and Fzmax2**	Maximum vertical and medial-lateral forces at the moment of propulsion
	**Fymin and Fzmin**	Minimum vertical and medial-lateral forces
	**GG Fymax1 and Fzmax1**	GG for maximum vertical and medial-lateral forces at ground contact
	**GG Fymax2 and Fzmax2**	GG for maximum vertical and medial-lateral forces at the moment of propulsion
	**Impulse after 50 ms**	Impulse at 50 ms after ground contact
	**Impulse after 75 ms**	Impulse at 75 ms after ground contact

GG = growth gradient.

### Statistical analysis

The biomechanical variables studied were initially analyzed to verify the statistical data distribution through the Shapiro-Wilks W test, to identify non-parametric data. The variables were described for each trial condition in terms of their central trend measurement and error: mean and standard deviation. For variables originating from the questionnaire, the data for each trial condition were shown as percentages.

The ground reaction force and temporal variables did not show normal distribution, and therefore the three trial conditions were compared by means of the Kruskal-Wallis non-parametric inferential test, using the Mann-Whitney test as a post-hoc test, in which the latter was strong enough to prove individual differences. Differences with a significance level (p) lower than 0.05 were considered significant.

The Excel (Microsoft) and Statistica v.5.1 (Statsoft Inc.) software were used for handling the statistical treatment, while Origin v.5.0 (*Microcal Software*) software was used for the mathematical treatment of ground reaction force variables.

## RESULTS

Table 2 shows the means, standard deviations and percentage distributions of the demographic and basketball-related variables for the subjects evaluated in this study.

**Table 2:** Means, standard deviations and percentage distributions of demographic variables, and description of basketball skill levels of subjects evaluated

Variables	Test group (n = 8)
**Age (years)**	22.4 ± 1.7
**Weight (kg)**	78.8 ± 9.1
**Height (m)**	1.9 ± 0.1
**Body mass index (kg/m^2^)**	21.8 ± 1.7
**Time playing basketball (years)**	10.8 ± 2.8
**Training session time (hours)**	2 ± 0
**Weekly training frequency (times/week) (median)**	3
**Bracing and taping usage (%)**	50

In this study all subjects were male, with a mean age of 22.4 ± 1.7 years. The subjects had a mean body mass index of 21.8 ± 2.7 kg/m^2^. The length of time for which they had participated in basketball was 10.8 ± 2.8 years. The mean frequency of training sessions was 3 ± 1 times per week, with a mean duration of two hours. With regard to the position they played, only two players were guards, four played as centers and seven as forwards, and of the eight subjects, five played in more than one position. A large majority of these subjects (87.5%, or 7/8) played basketball within the adult ranking while only one (12.5%) played within the 17-year-old ranking. Concerning their basketball skill levels, one player (12.5%) played professionally, one player (12.5%) played at the amateur level and the other 75% (6/8) played college basketball.

Fifty percent of the players use regularly external devices or taping during games and training sessions, yet 75% of the players had already suffered ankle sprains and, of these, 66% had injured both ankles.

**Figure 3:**
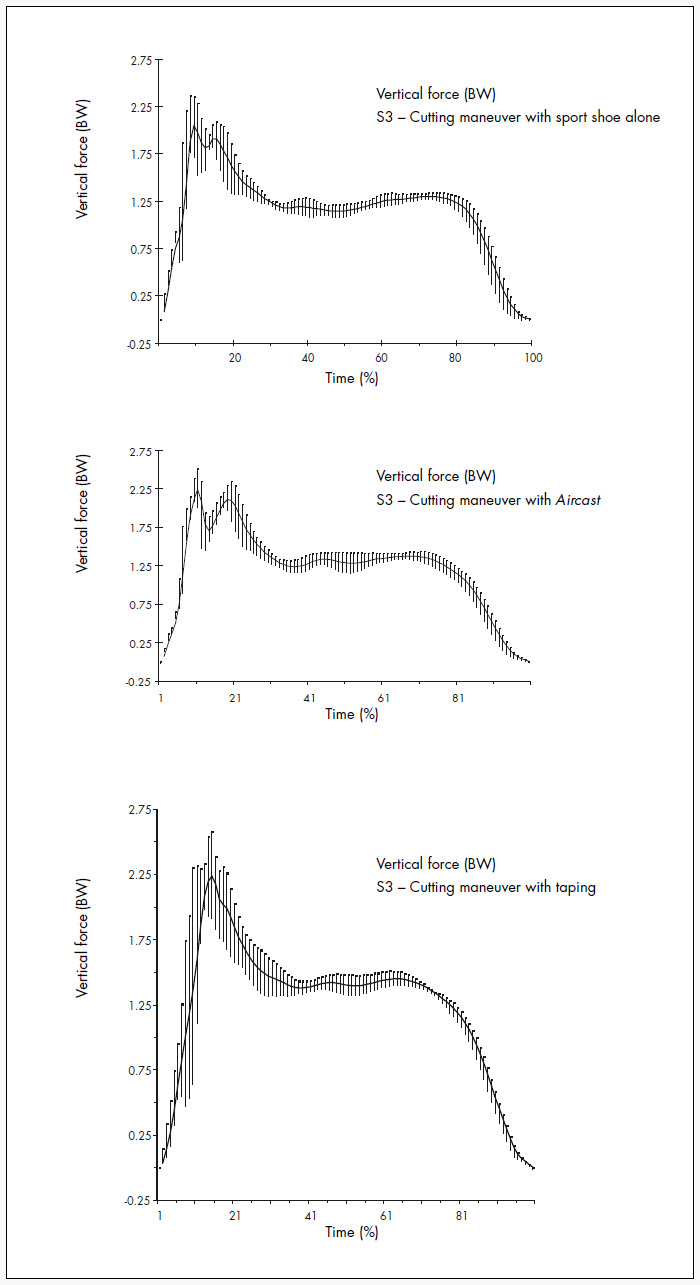
Graphic representation of mean and standard deviation for vertical ground reaction force during cutting maneuver in basketball with sport shoe alone, *Aircast* and taping for subject 3. BW = body weight.

Figure 3 and Table 3 show the mean and standard deviations for vertical ground reaction force variables during cutting maneuvers under the three trial conditions. For the sport shoe condition, the second peak force (Fymax2) was significantly higher than for the taping and *Aircast* conditions (p = 0.0000), but there was no difference in cutting maneuvers between the taping and *Aircast* conditions (p > 0.05). With regard to the Fymax2 growth gradient, there were significant differences between the three conditions (p = 0.0490), in which the value found for the sport shoe condition was higher than the values for *Aircas*t (p = 0.0020) and taping (0.0121). The gradient values for the taping and *Aircast* conditions did not show any statistical difference between them (p = 0.8778).

**Table 3:** Mean and standard deviation for vertical ground reaction force during cutting maneuver in basketball, with sport shoe alone, taping and *Aircast* ankle bracing (n = 8)

Variables	Sport shoe alone	*Aircast*	Taping	p
**Fymax1 (BW)**	2.04 ± 0.37	2.03 ± 0.43	2.11 ± 0.43	> 0.05
**Fymax2 (BW)**	2.02 ± 0.26	1.44 ± 0.13	1.50 ± 0.17	0.0000
**Fymin (BW)**	1.20 ± 0.19	1.16 ± 0.20	1.14 ± 0.25	> 0.05
**GG Fymax1 (BW/s)**	45.64 ± 22.06	41.01 ± 16.92	44.91 ± 21.54	> 0.05
**GG Fymax2 (BW/s)**	29.20 ± 27.05	15.85 ± 10.65	18.64 ± 15.59	0.0490
**Impulse after 50 ms (N.s)**	0.05 ± 0.01	0.05 ± 0.01	0.05 ± 0.02	> 0.05
**Impulse after 75 ms (N.s)**	0.09 ± 0.02	0.08 ± 0.02	0.09 ± 0.02	> 0.05

GG = growth gradient; BW = body weight; N.s = Newton.seconds; Fymax1 = maximum vertical and medial-lateral forces at foot contact on the ground; Fymax2 = Maximum vertical and medial-lateral forces at the moment of propulsion; Fymin = Minimum vertical and medial-lateral forces; GG Fymax1 = GG for maximum vertical and medial-lateral forces at ground contact; GG Fymax2 = GG for maximum vertical and medial-lateral forces at the moment of propulsion.

**Figure 4:**
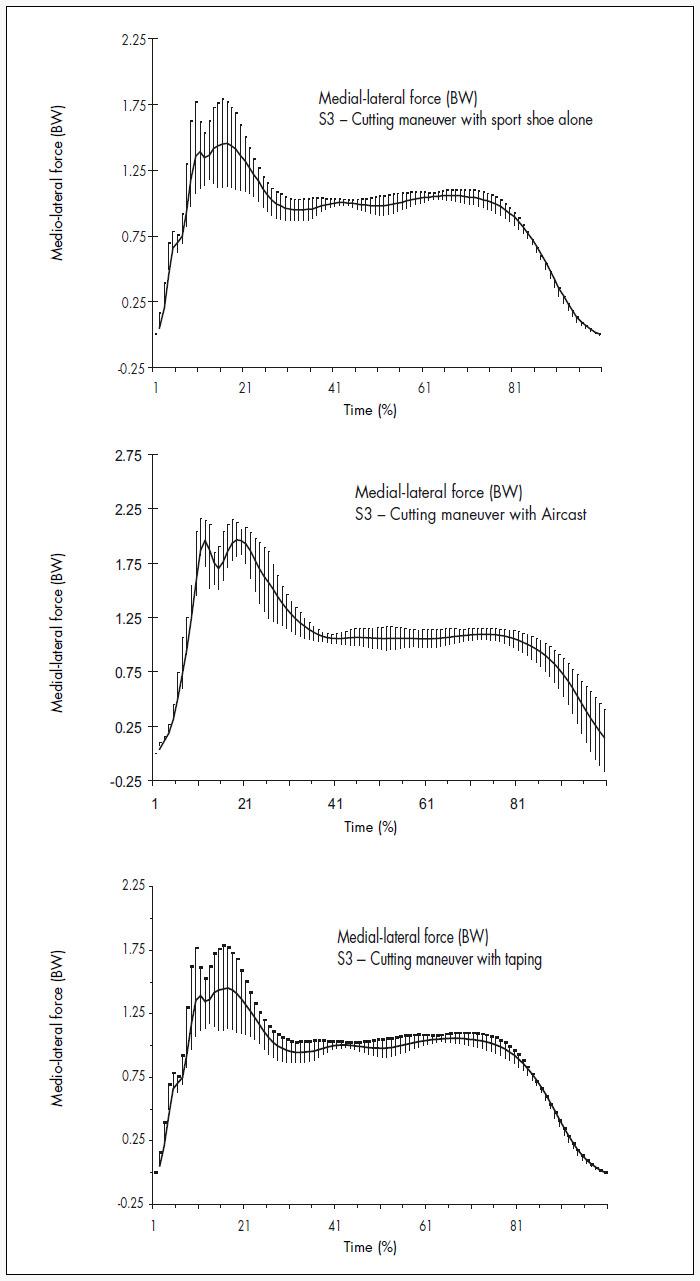
Graphic representation of mean and standard deviation for horizontal ground reaction force during cutting maneuver in basketball with sport shoe alone, *Aircast* and taping for subject 3. BW = body weight.

Figure 4 and Table 4 show the mean and standard deviation of medial-lateral ground reaction force variables during cutting maneuvers while using bracing, taping and sport shoes alone. There were no significant differences with respect to force magnitudes between these three conditions. However, the Fzmax1 growth gradient was significantly lower for the sport shoe condition than for the taping condition (p = 0.0433).

**Table 4:** Mean and standard deviation of horizontal ground reaction force during cutting in basketball with and without ankle bracing: sport shoe, taping and *Aircast* (n = 8)

Variables	Sport shoe alone	*Aircast*	Taping	p
**Fzmax1 (BW)**	1.53 ± 0.35	1.54 ± 0.39	1.54 ± 0.42	> 0.05
**Fzmax2 (BW)**	0.85 ± 0.40	0.95 ± 0.26	1.00 ± 0.31	> 0.05
**Fzmin (BW)**	0.79 ± 0.12	0.82 ± 0.21	0.81 ± 0.19	> 0.05
**GG Fzmax1 (BW/s)**	27.36 ± 11.19	30.73 ± 11.01	35.38 ± 16.78	0.0433
**GG Fzmax2 (BW/s)**	10.75 ± 10.61	11.44 ± 11.56	14.43 ± 16.19	> 0.05
**Impulse 50 ms (N.s)**	0.03 ± 0.01	0.03 ± 0.01	0.03 ± 0.01	> 0.05
**Impulse 75 ms (N.s)**	0.07 ± 0.02	0.02 ± 0.02	0.07 ± 0.02	> 0.05

GG = growth gradient; BW = body weight; N.s = Newton.seconds; Fymax1 = maximum vertical and medial-lateral forces at foot contact on the ground; Fymax2 = Maximum vertical and medial-lateral forces at the moment of propulsion; Fymin = Minimum vertical and medial-lateral forces; GG Fymax1 = GG for maximum vertical and medial-lateral forces at ground contact; GG Fymax2 = GG for maximum vertical and medial-lateral forces at the moment of propulsion.

## DISCUSSION

The mean age in this study (22 years) shows that the subjects were all young individuals, which lowered the likelihood of chronic diseases related to decreased mobility. A large majority of the subjects (87.5%) played basketball within an adult ranking, at college level. Moreover, all the subjects had negative results from clinical tests for functional and mechanical instability, thus demonstrating that none of them had any kind of ankle joint instability that could alter the results.

The mean body mass index (BMI) was 21.8 kg/m^2^, which is within normal limits (i.e. between 20 and 25),^27^ thereby eliminating the possibility that there might have been excessive loading on the joints, or other abnormalities resulting from states of excessive weight.

A considerable percentage of the subjects (50%) were regularly using external devices or taping during games and training sessions as a way of preventing ankle sprains. Yet we observed in this study that most of the players (75%) had already suffered ankle sprains and, of these, 66% had injured both ankles. This demonstrates that these are very common basketball injuries, and that there is therefore a need to improve the knowledge of how to prevent this kind of injury, and how such injuries are related to the types of maneuvers made by players.

A study by McKay et al.^28^ did not find any correlations for gender, age, mass, height and training frequency versus the incidence of ankle injuries. However, it is important to note that the sport of basketball typically presents training overload and this was the case for the players evaluated in that study. Such training overload would increase the musculoskeletal loading and, consequently, place stress on ankles and favor the incidence of injuries.

One study analyzed ground reaction force in lateral displacement and found no differences between control conditions and conditions using orthoses and taping.^9^ However, in the present study there were statistically significant differences in the vertical and medial-lateral components of the ground reaction force.

In considering the cutting maneuver, it was observed that under the taping conditions, the vertical force magnitude (Fymax1) at the moment of foot contact with the ground tended to be higher than under the conditions of sport shoes alone and *Aircast*, and that the growth gradient (GG) for this force (GG Fymax1) tended to be higher solely in relation to *Aircast*. Although taping produced higher vertical force in relation to the sport shoe alone, the GG values for both conditions remained similar. That is, taping increased the vertical peak force, but over a proportionally longer time interval. The consequent interpretation for this is that the excessive loading under this condition did not change. In relation to *Aircast*, taping tended to produce a higher vertical force value over a shorter time interval during ground impact. The growth gradient for vertical force under the taping condition had higher inclination than for *Aircast*, thus indicating that this bracing was exerting a better shock-absorbing effect than were the sport shoe alone and taping, and consequently, less loading on the more distal joints.

From analysis of the medial-lateral component of the cutting maneuver, it was found that, for taping, the growth gradient for this force at ground contact (GG Fzmax1) was also significantly higher than for the sport shoe alone (p = 0.0433) and tended to be higher than for *Aircast*, although the medial-lateral force values were similar. This result is in agreement with the earlier study by Cordova et al.,^9^ which did not find any differences in impact peak force magnitude (foot contact with the ground) for the medial-lateral component during lateral displacements that were similar to cutting maneuvers. However, these authors did not analyze the time needed to reach this peak force at the moment of ground contact. The increased GG Fzmax1 under the taping condition that was observed in the present study means that the use of this stabilizing technique decreased the time to reach the medial-lateral peak force. This higher growth gradient for medial-lateral force may result from a more rigid ankle, which would have smaller inversion and eversion movement amplitudes as a result of taping.

On the other hand, a high GG Fzmax1 value may signify more excessive loading on the ankle and lower limb joints over a medium and long-term basis. Bracing usage would limit the time for external forces to act on the small movement amplitude available for the joint, thus making the other foot and ankle structures absorb greater forces.^9^ Furthermore, higher compressive forces might be generated, thereby resulting in greater injury risk. Although the *Aircast* and taping conditions did not show any statistical difference, these conditions showed a tendency towards higher medial-lateral peak force on the impact and propulsion moments (Fzmax1 and Fzmax2) and greater growth rates for these forces. As previous stated by Riemann et al.,^15^ these alterations indicate that during dynamic activities the musculoskeletal structures of the body may be subjected to loads within shorter time periods.

At the propulsion phase of the cutting maneuver, the sport shoe condition showed significantly higher vertical force values (Fymax2) than did the taping (p = 0.0000) and *Aircast* (0.0000) conditions. The force growth gradient (GG Fymax2) was also significantly higher than for taping (p = 0.0121) and *Aircast* (p = 0.0020). Bracing produced smaller vertical forces over longer time intervals, i.e. after the minimum vertical force moment (Fymin) the vertical GRF peaks for impulsion were reached in less time for taping and *Aircast*, in comparison with the sport shoe. But the taping and *Aircast* conditions were shown to be significantly similar. As mentioned earlier, it can be interpreted that bracing might be reducing the excessive loading on the vertical joint at the moment of impulsion during the cutting maneuver, a situation that the sport shoe alone was unable to implement. This finding is in agreement with the study by Anderson et al.,^29^ which suggested that the use of non-rigid orthoses slows the inversion movement. Therefore, the musculoskeletal system might have more time to respond to the demands from external forces, thereby generating less excessive loading on the foot and ankle structures.

Concerning the medial-lateral force at the propulsion phase, the taping condition tended to show a higher peak force value in comparison to the *Aircast* condition, and the latter was higher than for the sport shoe condition. The growth gradient for taping also tended to be higher than for the other two conditions. It can be considered that, when taping is used, at the moment of propulsion there are more foot movement instabilities, which is unexpected, since this bracing should serve to stabilize medial-lateral maneuvers.

In summary, at the moment the ground receives the load, taping increased the vertical force in relation to the sport shoe alone, but over a longer time interval, without increasing excessive ankle loading. On the other hand, in relation to medial-lateral force, the taping condition reached a peak in less time, which might generate greater inversion/eversion loading on the player's foot. In this same maneuver, the *Aircast* exerted better shock-absorbing effect than did the other two conditions, since it generated less vertical force over a longer time interval and smaller medial-lateral forces than did taping. With regard to propulsion during the cutting maneuver, bracing reduced the magnitude and increased the time needed to reach vertical peak force, thereby allowing more time for adjustments and adaptations of the muscle control system. But for medial-lateral force, taping produced higher peak forces in less time than did the other two conditions, thereby generating more excessive loading during inversion/eversion.

The search for decreasing the mediallateral forces is leading towards an increase in other GRF components, i.e., increases in the vertical components of this force, for example. According to Cordova et al.,^9^ this would cause increased compressive forces on the skeletal system. These alterations indicate that during dynamic activity the musculoskeletal structures of the body may be subjected to loads within shorter time periods. Whether these effects are detrimental over time remains speculative at this point and requires further research.

## FINAL CONSIDERATIONS

The effects of prophylaxis on sprain prevention and the use of such measures for sprain treatment have already been comprehensively described in the literature. However, few studies have analyzed ground reaction forces during dynamic activities with ankle bracing or taping, and most of these did not find differences between control and study conditions.

One alternative to using ankle bracing or taping for preventing injuries would be muscle and proprioception training to improve neuromuscular response during ankle mobilization. Increasing flexibility would allow the ankle to reach a certain movement amplitude without reaching the limit at which injury occurs; increasing muscle strength would allow muscles to resist the maneuver that might result in ankle sprains; decreasing neuromuscular time response would allow an individual to react more quickly to a possible injury.^30^

Several studies in the literature have given ambiguous evidence concerning the effects of bracing and taping on GRF, performance, balance and muscle activity.^30^ The mechanisms by which external ankle devices and taping act are still unclear and more studies are needed in order to understand their effects on sporting activities. It is therefore suggested that coaches and physical therapists should be careful in prescribing bracing devices or taping for healthy athletes.

## CONCLUSIONS

In the present study, it was observed that bracing and taping generated alterations in vertical and medial-lateral ground reaction forces between taping, *Aircast* and control conditions. As expected, bracing and taping attenuated vertical or mediallateral ground reaction force components in some instances, while on the other hand increasing others, which would lead to increased compressive and inversion/eversion forces on the skeletal system by restricting joint mobility. Therefore, although players frequently use bracing devices or taping, whether these effects are detrimental over time also remains speculative at this point and requires further research.
